# Gamma-linolenic acid inhibits both tumour cell cycle progression and angiogenesis in the orthotopic C6 glioma model through changes in VEGF, Flt1, ERK1/2, MMP2, cyclin D1, pRb, p53 and p27 protein expression

**DOI:** 10.1186/1476-511X-8-8

**Published:** 2009-03-17

**Authors:** Juliano Andreoli Miyake, Marcel Benadiba, Alison Colquhoun

**Affiliations:** 1 Department of Cell and Developmental Biology, Biomedical Sciences Institute, University of São Paulo, São Paulo, SP, Brazil

## Abstract

**Background:**

Gamma-linolenic acid is a known inhibitor of tumour cell proliferation and migration in both *in vitro *and *in vivo *conditions. The aim of the present study was to determine the mechanisms by which gamma-linolenic acid (GLA) osmotic pump infusion alters glioma cell proliferation, and whether it affects cell cycle control and angiogenesis in the C6 glioma *in vivo*.

**Methods:**

Established C6 rat gliomas were treated for 14 days with 5 mM GLA in CSF or CSF alone. Tumour size was estimated, microvessel density (MVD) counted and protein and mRNA expression measured by immunohistochemistry, western blotting and RT-PCR.

**Results:**

GLA caused a significant decrease in tumour size (75 ± 8.8%) and reduced MVD by 44 ± 5.4%. These changes were associated with reduced expression of vascular endothelial growth factor (VEGF) (71 ± 16%) and the VEGF receptor Flt1 (57 ± 5.8%) but not Flk1. Expression of ERK1/2 was also reduced by 27 ± 7.7% and 31 ± 8.7% respectively. mRNA expression of matrix metalloproteinase-2 (MMP2) was reduced by 35 ± 6.8% and zymography showed MMP2 proteolytic activity was reduced by 32 ± 8.5%. GLA altered the expression of several proteins involved in cell cycle control. pRb protein expression was decreased (62 ± 18%) while E2F1 remained unchanged. Cyclin D1 protein expression was increased by 42 ± 12% in the presence of GLA. The cyclin dependent kinase inhibitors p21 and p27 responded differently to GLA, p27 expression was increased (27 ± 7.3%) while p21 remained unchanged. The expression of p53 was increased (44 ± 16%) by GLA. Finally, the BrdU incorporation studies found a significant inhibition (32 ± 11%) of BrdU incorporation into the tumour *in vivo*.

**Conclusion:**

Overall the findings reported in the present study lend further support to the potential of GLA as an inhibitor of glioma cell proliferation *in vivo *and show it has direct effects upon cell cycle control and angiogenesis. These effects involve changes in protein expression of VEGF, Flt1, ERK1, ERK2, MMP2, Cyclin D1, pRb, p53 and p27. Combination therapy using drugs with other, complementary targets and GLA could lead to gains in treatment efficacy in this notoriously difficult to treat tumour.

## Background

Gamma-linolenic acid has been proposed as an antitumour therapy and has proven efficacy in several tumour types [1,2]. Studies have used GLA for the treatment of human gliomas although these trials were preliminary in nature [3-5]. Studies in the C6 rat glioma model have shown GLA inhibits cell proliferation and induces apoptosis and similar results have been obtained with human glioma cells in primary culture [6-9]. GLA is known to induce reactive oxygen species generation and cause lipid peroxidation in tumour cells and leads to altered mitochondrial metabolism and ultrastructure, cytochrome c release, caspase activation and apoptosis [10-14]. Both GLA and its metabolic products can alter the gene expression of several proteins and GLA is known to inhibit glioma cell migration [14-16].

One of the major problems of glioma progression is intense angiogenesis which has been related not only to tumour nutrition but also to tumour cell migration along the basement membrane of the growing blood vessels [17]. In gliomas, the best characterized pro-angiogenic factor is vascular endothelial growth factor (VEGF), whose overexpression is correlated with increasingly malignant phenotypes [18]. VEGF and its receptors Flt1 (VEGFR1) and Flk1 (VEGFR2) are important proteins in the angiogenic process and represent a treatment target in many tumours including gliomas. In order for angiogenesis to progress extracellular matrix (ECM) degradation is necessary and the metalloproteinases 2 and 9 (MMP2 and MMP9) are highly expressed in gliomas [19,20]. Interestingly, GLA is known to inhibit both endothelial cell proliferation and induce endothelial cell apoptosis. Several studies have reported GLA-induced changes in endothelial cells including altered occludin and VE-cadherin expression and altered barrier properties [21,22]. Polyunsaturated fatty acids have also been reported to influence MMP2 expression in endothelial cells [23]. However the effects of GLA on cell cycle and angiogenesis related proteins in gliomas *in vivo *has not been explored and is the principal focus of the present study.

The aim of the study was to determine the effects of slow (0.5 μl/hr) osmotic pump infusion of 5 mM GLA on factors related to the angiogenic process and to the control of the cell cycle in the C6 rat glioma model. The mRNA and protein expression were studied of (i) angiogenesis related proteins: vascular endothelial growth factor (VEGF), VEGF receptors Flt1 and Flk1, matrix metalloproteinase 2 (MMP2), matrix metalloproteinase 9 (MMP9), ERK1 and ERK2, and (ii) cell cycle control related proteins: pRb, cyclin D1, E2F1, p16, p21, p27 and p53. Immunohistochemical localization of proteins was performed by light microscopy and semi-quantitative analysis of protein expression was performed for VEGF, Flt1 and Flk1. MMP2 proteolytic activity was determined by zymography in order to determine GLA effects on ECM degradation capacity *in vivo*. Glial fibrillary acidic protein (GFAP) was immunohistochemically localized in order to determine the tumour area by image analysis. Bromodeoxyuridine (BrdU) incorporation was analysed by immunohistochemical localization of the compound to determine the effects of GLA on S-phase DNA synthesis *in vivo*.

## Methods

### Cell culture

C6 rat glioma cells were obtained from the ATCC and stocks were maintained frozen (liquid nitrogen) in Dulbecco's modified Eagle's medium (DMEM) supplemented with 10% foetal calf serum and 20% glycerol. Stock cells were grown in DMEM containing 10% foetal calf serum and antibiotics (penicillin 50 U/ml, streptomycin 50 μg/ml). Cells in the exponential phase of growth were used, growing in 75 cm^2 ^flasks in a humidified atmosphere of 5% CO2: 95% air at 37°C.

### Surgical procedures

C6 rat glioma cells were grown in Dulbecco's modified Eagle medium (DMEM) containing 10% foetal calf serum and antibiotics (penicillin/streptomycin). Cells in the exponential phase of growth were used and a suspension prepared in sterile saline at a concentration of 5 × 10^5 ^cells per 4–5 μl. Adult female Wistar rats of 250–350 g (n = 34) were anaesthetised with an intramuscular injection of ketamine:xylazine, 10 mg:1.5 mg/100 g body weight to provide deep anaesthesia and analgesia. The rats were placed on a stereotaxic surgical table, a midline incision was made and a burrhole was drilled 0.48 mm anterior and 3 mm lateral to bregma. The C6 cell suspension was slowly injected into the striatum using a Hamilton syringe at a depth of 5.4 mm to the bone surface and the needle left *in situ *for 3 minutes before its removal. After 14 days Alzet osmotic pumps containing artificial cerebrospinal fluid (CSF) (Alzet) or 5 mM GLA in artificial CSF were surgically implanted and attached to Alzet brain infusion kits. Artificial CSF was chosen as a vehicle solution in order to mimic more closely the composition of the interstitial fluid within the brain. These concentrations were chosen based on previous work and unpublished data from our laboratory [7]. The pump infusion rate was 0.5 μl/hr with a duration of 2 weeks. After a further 14 days the rats were killed by transcardiac perfusion with 4% formaldehyde in 0.1 M phosphate buffer, pH7.4 or by anaesthetic overdose for removal of fresh tissues for RT-PCR and western blotting. This procedure was approved by the Ethical Commission for Animal Experimentation of the Biomedical Institute (University of São Paulo) – protocol number 190/02.

### Antibodies

The antibodies used in this study were: MMP-2 (goat), MMP-2 (mouse), MMP-9 (goat), MMP-9 (mouse), VEGF (mouse), VEGF (rabbit), Flt-1 (rabbit), Flk-1 (rabbit), cd34 (mouse), BrdU (mouse), pRb (rabbit), cyclin D1 (rabbit), E2F1 (rabbit), p16 (mouse), p21 (rabbit), p27 (rabbit), p53 (rabbit), ERK1/ERK2 (rabbit) and GFAP (goat). All of the primary antibodies were purchased from Santa Cruz Biotechnology, USA. Biotinylated Ulex europaeus lectin was from Vector Laboratories, USA. Biotinylated secondary antibodies (anti-goat, anti-mouse and anti-rabbit) used for IHC were produced in donkey (Santa Cruz Biotechnology, USA), and the streptavidin-biotin/HRP (horseradish peroxidase) was produced by Amersham Biosciences, UK.

### Immunohistochemical (IHC) analysis

The perfused brains were cryoprotected in a solution of 20% sucrose in 0.1 M potassium phosphate buffer (KPB) overnight. The brain sections were cut on a freezing microtome (Leica SM 2000R) and mounted on gelatinized slides. The sections were dried at 40°C-50°C for 2 hours and were maintained at -20°C until analysis. Immunohistochemical analysis followed [14] described briefly here. The sections were incubated at room temperature overnight with the respective primary antibody (MMP-2, MMP-9, Flt-1 and Flk-1, 1:100, VEGF, CD34 and BrdU 1:200 and GFAP, 1:500) diluted in PBST. The negative controls received only PBST. The slides were washed with PBST and incubated with the secondary antibodies (1:500–1:1000 in PBST) for 90 minutes. The slides were washed again with PBST and incubated with streptavidin-HRP (1:100–1:200 in PBST) for 60 minutes. The reactions were developed with 0.04% 3,3'-diaminobenzidine (DAB) + 0.03% H_2_O_2_. For MMP-2, Flt-1, Flk-1 and GFAP the DAB reactions were intensified with an OsO_4 _solution (0.04%) for 30 minutes. All slides were counterstained with 0.1% methyl-green, dehydrated and mounted with Permount^® ^(Fisher Scientific).

### mRNA expression analysis by RT-PCR

Samples were dissected with the aid of a surgical microscope and used for total RNA extraction with Trizol (Life Technologies, USA). The first strand of complementary DNA (cDNA) was generated from 1 μg RNA as previously described [24]. PCR amplification cycle: 1 minute at 94°C, 1 minute at primer-specific temperature and 1 minute at 72°C. To ensure the exponential phase of amplification, the number of PCR cycles was determined and optimized for each of the proteins. Controls for non-specific amplification showed no bands on gel (data not shown). Semi-quantitative gene expression data was calculated by the ratio of respective gene/GAPDH density after ethidium bromide staining. GAPDH expression remained unchanged after GLA exposure. Primers used were as follows (sense/antisense):

MMP-2 (500 bp):5'-TGGCAGTGCAATACCTGAAC-3',

5'-CAAGGTCCATAGCTCATCGTC-3';

MMP-9 (531 bp):5'-GAGGAATACCTGTACCGCTATG-3',

5'-CAAACCGAGTTGGAACCAC-3';

VEGF (148 bp):5'-CTGTACCTCCACCATGCCAAG-3',

5'-GGTACTCCTGGAAGATGTCCACC-3';

FLT-1 (453 bp):5'-TGGAAGGAGGCGAGGATTACAGTGAGA-3',

5'GGTAGATTCCAGGTGTGGCATACTCTGGTG-3';

FLK-1(445 bp):5'-GTACTCCAGCGACGAGGCAGGACTTTTA-3',

5'-TTTTATCCAGTTTCACAGAGGGCTCCATTG-3';

GAPDH (306 bp):5'-GTCGGTGTGAACGGATTTG-3',

5'-ACAAACATGGGGGCATCAG-3';

ERK1 (188 bp) 5'-CCTGCTGGACCGGATGTTA-3'

5'-GTCTCTTGGAAGATCAGCTC-3'

pRb (549 bp): 5'-TCTACCTCCCTTTCCCTGTTT-3',

5'-AGTCATTTTTGTGGGTGTTGG-3';

p16 (180 bp): 5'-TCTGCAGATAGACTAGCCA-3',

5'-CTCGCAGTTCGAATCTGCA-3';

p21 (200 bp): 5'-TCCGATCCTGGTGATGTCC-3',

5'-CGAACACGCTCCCAGACGT-3';

p27 (325 bp): 5'-GCAGCTTGCCCGAGTTCTAC-3',

5'-TTCTTGGGCGTCTGCTCCAC-3';

p53 (271 bp): 5'-GTGGCCTCTGTCATCTTCCG-3',

5'-CCGTCACCATCAGAGCAACG-3';

E2F1 (143 bp): 5'-ACGCTATGAAACCTCACTAAA-3',

5'-AGGACATTGGTGATGTCATA-3';

Cyclin D1 (435 bp): 5'-TGTTCGTGGCCTCTAAGATGA-3',

5'-GCTTGACTCCAGAAGGGCTT-3'.

### Western blotting

20-μg sample proteins were electrophoresed through 7.5% SDS-PAGE and transferred to nitrocellulose membranes (Hybond ECL membrane, Amersham Pharmacia Biotech). Immunoblotting followed standard methods [9] and fluorescent bands (Alexa-488 label) were visualized in an image system (Molecular Dynamics Typhoon 8600 Variable Mode Imager). The secondary antibodies conjugated with Alexa-488 (anti-goat, anti-mouse and anti-rabbit) used were produced in donkey (Invitrogen – Molecular Probes).

### BrdU incorporation

DNA synthesis was determined *in vivo *by administration of 100 mg/kg BrdU intraperitoneally 90 minutes before transcardiac perfusion of CSF or GLA + CSF treated rats. Immunohistochemical detection of cells containing BrdU was performed on cryosections as described above. The method used followed doses used in mouse studies [25].

### Zymography

The activity of MMP2 and MMP9 was detected using zymography on a 10% SDS-PAGE gel containing 1 mg/ml gelatin. The proteolytic activity was identified as clear bands on a blue background after Coomassie blue staining of the gel after protein separation by electrophoresis and incubation for 20 hrs following the method of [26]. The intensity of the bands was determined by densitometry and the activity was attributed to MMPs as it was readily inhibited by 5 mM EDTA solution during development.

### Gas Chromatography-Mass Spectrometry Analysis

Lipids were extracted and fatty acid methyl esters were formed by the sulphuric acid/anhydrous methanol method [27,28]. The fatty acid methyl esters were separated on a DB-23 column [(50% cyanopropyl)methyl polysiloxane, 0.25 μm film thickness, 0.250 mm × 60 m], in a Shimadzu GCMS model QP5050 as described in [14]. Individual fatty acid methyl esters were identified by comparison with authentic standard retention times and mass spectra.

### Experimental Analyses

Immunohistochemistry images were captured and analysed using a CCD camera, an Olympus Optiphot microscope and Image ProPlus software. All labelling above background intensity was selected and a mask created for immunolabelling area calculations on calibrated images. Values were expressed in μm^2 ^± SEM and are the mean of 6–10 random areas within each tumour section for each experimental animal, totalling 664 areas analysed for this experiment.

The microvessel density was calculated by counting 5 random high power fields (×40 objective) in each tumour section for each experimental animal, totalling 145 areas analysed for this experiment. Vessels of less than 14 μm were considered as microvessels, following criteria from [29].

Tumour area was estimated by two parameters the first being area analysis after image capture of GFAP labelled tumour sections. The second method was by calculating the rostrocaudal extension of the tumour (length) from the number of cryosections obtained for immunohistochemical analysis multiplied by section thickness. Image analysis was performed using Sigma Scan and Image Pro Plus software.

The semi-quantitative analysis of RT-PCR products and quantitative analysis of Western blots was performed using a Molecular Dynamics Typhoon 8600 Variable Mode Imager and ImageQuant. All data are presented as the mean ± SEM. Statistical differences were determined by one-way ANOVA with *post-hoc *Tukey's test and p < 0.05 was considered significant.

## Results

**Figure 1:**
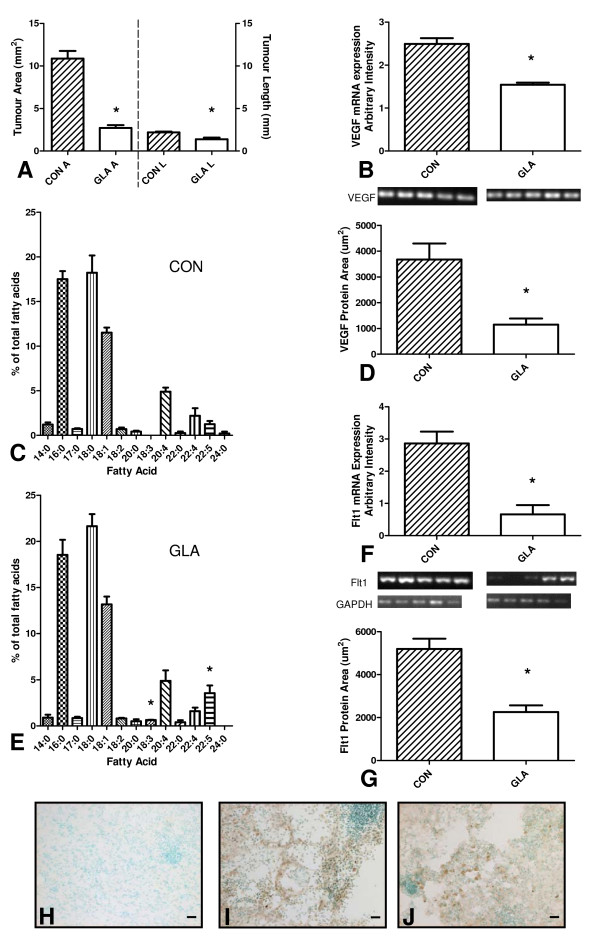
**Effect of 14-day osmotic pump infusion of 5 mM gamma-linolenic acid (GLA) or control cerebrospinal fluid (CON = CSF) on C6 glioma development *in vivo***. (A) Tumour size estimated by glial fibrillary acidic protein labeling and image analysis with ImageProPlus. Average tumour area (mm^2^) and length = rostral-caudal extension (mm) were plotted for CON (control CSF) and GLA (5 mM GLA + CSF) (n = 12), statistical significance, * = p < 0.0001. Left side of graph presents area (CON A and GLA A) while right side of graph presents length (CON L and GLA L). (B and F) mRNA expression of VEGF and Flt-1 in CON and GLA-treated tumours, with GAPDH as an internal control (n = 10), statistical significance, * = p < 0.001. (D and G) Immunohistochemical labeling for VEGF and Flt-1 CON and GLA-treated tumours, semi-quantitative analysis with ImageProPlus. (n = 9), statistical significance, * = p < 0.01 (C and E) Fatty acid composition by gas chromatography-mass spectrometry of CON (C) and GLA (E) treated tumours (n = 11), statistical significance, * = p < 0.0001. (H-J) Representative images of control (H), VEGF (I) and Flt-1 (J) immunohistochemical labeling (n = 9).

The infusion of 5 mM GLA in CSF caused a significant decrease in C6 tumour growth in comparison with CSF alone *in vivo *(Figure 1A), as previously reported at lower concentrations [7]. The average tumour area was reduced by 75 ± 8.8% while the tumour length (rostrocaudal extension) was reduced by 38 ± 9.7%. Tumour fatty acid composition was also altered by GLA treatment with significant increases in 18:3, n-6 and 22:5, n-6 content (Figure 1E).

After confirmation of the efficacy of treatment the expression of angiogenesis related proteins was examined by mRNA and/or protein expression analysis. The mRNA and protein expression of VEGF and its receptors Flt1 and Flk1 were compared in control CSF and 5 mM GLA treated animals. In Figure 1B, 1D the mRNA expression of VEGF was reduced by 38 ± 5.8% in the presence of 5 mM GLA while the protein expression was reduced by 71 ± 16%. The mRNA expression of Flt1 was reduced by 77 ± 16% in the presence of 5 mM GLA while the protein expression was reduced by 57 ± 5.8% (Figure 1F, 1G). While GLA increased the mRNA expression of Flk1 by 39 ± 12% its protein expression was unchanged in the GLA treated tumour (data not shown).

**Figure 2:**
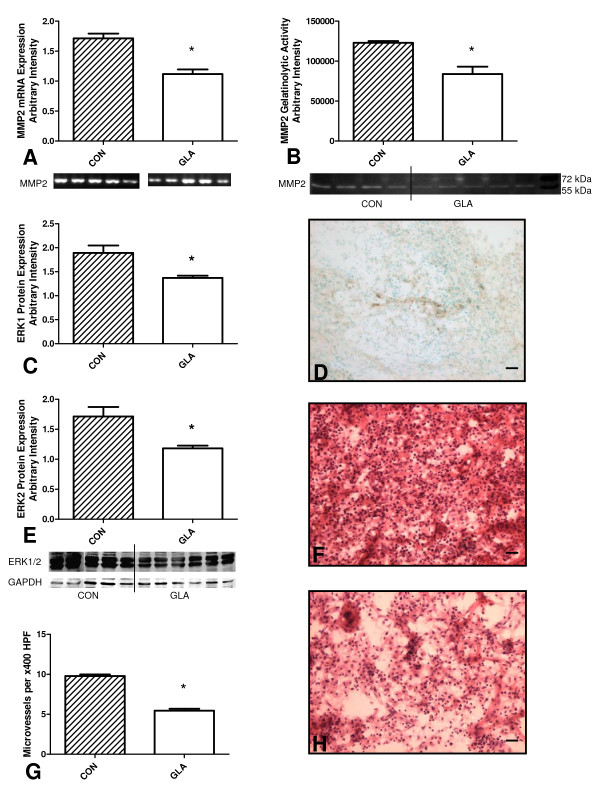
**Effect of 14-day osmotic pump infusion of 5 mM gamma-linolenic acid (GLA) or control cerebrospinal fluid (CON = CSF) on protein expression and angiogenesis in the C6 glioma *in vivo***. (A) mRNA expression of MMP2 in CON and GLA-treated tumours, with GAPDH as an internal control (n = 10), statistical significance, * = p < 0.001. (B) Zymographic detection of MMP2 proteolytic activity in CON and GLA-treated tumours (n = 9), statistical significance, * = p < 0.004. Lanes 1–4 CON, 5–9 GLA. (C and E) Western blot of ERK1 and ERK2 protein expression, with GAPDH as an internal control (n = 11), statistical significance, * = p < 0.006. Lanes 1–5 CON, 6–11 GLA. (D) Representative images of MMP2 immunohistochemical labeling (n = 9). (F and H) Haematoxylin and eosin stained sections of CON (F) and GLA (H) treated tumours. Note large number of blood vessels and tumour cells in the CON tumour in comparison with the GLA treated tumour. (G) Number of microvessels in CON and GLA-treated tumours (n = 9). Statistical significance, * = p < 0.001.

GLA infusion was associated with a decrease in MMP2 (35 ± 6.8%) mRNA expression (Figure 2A). MMP9 mRNA was not expressed by the tumour and this was confirmed by negative staining with MMP9 antibody on cryosections and lack of activity in zymograms (data not shown). Due to the heterogeneous distribution of MMP2 in the tumour tissue immunohistochemical quantification was not possible. Zymography confirmed a significant decrease (32 ± 8.5%) in proteolytic activity of MMP2 in the presence of GLA (Figure 2B). The presence of 5 mM GLA caused a significant decrease in both ERK1 (27 ± 7.7%) and ERK2 (31 ± 8.7%) protein expression (Figure 2C, 2E) and mRNA expression (data not shown).

In the knowledge that 5 mM GLA caused significant changes in mRNA expression and protein expression of important factors involved in the angiogenic process we further investigated these effects by measuring the microvessel density of treated and control tumours. The microvessel density of the GLA treated tumour was reduced by 44 ± 5.4% versus the control tumour (Figure 2G).

**Figure 3:**
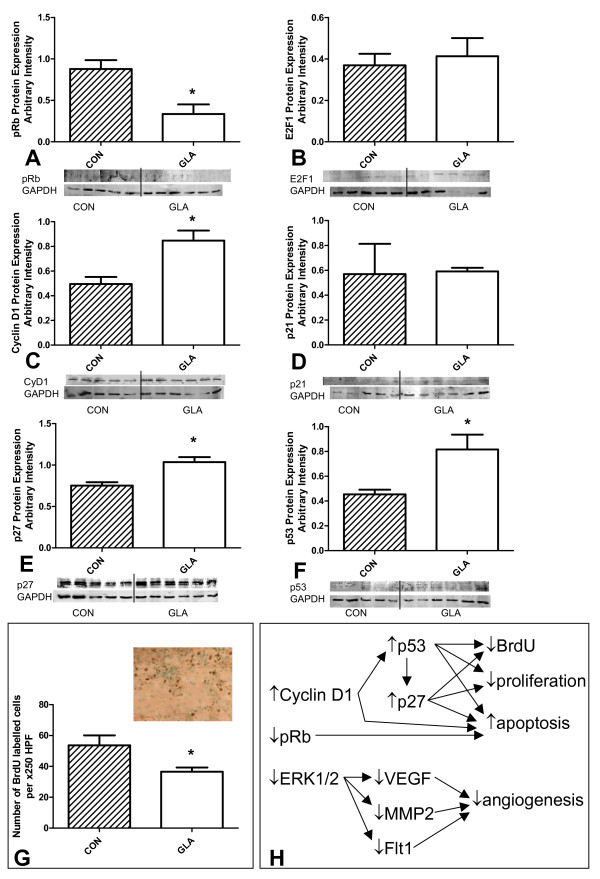
**Effect of 14-day osmotic pump infusion of 5 mM gamma-linolenic acid (GLA) or control cerebrospinal fluid (CON = CSF) on cell cycle control in the C6 glioma *in vivo***. (A) Western blot of pRb protein expression, statistical significance, * = p < 0.009. (B) Western blot of E2F1 protein expression. (C) Western blot of cyclin D1 protein expression, statistical significance, * = p < 0.008. (D) Western blot of p21 protein expression. (E) Western blot of p27 protein expression, statistical significance, * = p < 0.005. (F) Western blot of p53 protein expression, statistical significance, * = p < 0.026. For all Western blots GAPDH was used as an internal control and is presented below the protein bands of interest in A-F, (n = 11), Lanes 1–5 CON, 6–11 GLA. (G) Bromodeoxyuridine incorporation *in vivo*, representative image of labelled cells (n = 9), statistical significance, * = p < 0.015. (H) Schematic presentation of changes found in GLA-treated tumours in comparison with control tumours and possible interactions among proteins based on current literature.

GLA was found to alter the expression of several proteins involved in cell cycle control (Figure 3). pRb protein expression was decreased (62 ± 18%) while E2F1 remained unchanged (Figure 3A, 3B). Cyclin D1 protein expression was increased by 42 ± 12% in the presence of GLA (Figure 3C). The cyclin dependent kinase inhibitors p21 and p27 responded differently to GLA, p27 expression was increased (27 ± 7.3%) while p21 remained unchanged (Figure 3D, 3E). C6 glioma cells did not express the cyclin-dependent kinase inhibitor p16 (data not shown) as previously reported [30]. The expression of p53 was increased (44 ± 16%) by GLA (Figure 3F). Finally, the BrdU incorporation studies found a significant inhibition (32 ± 11%) of BrdU incorporation into the tumour *in vivo *(Figure 3G).

## Discussion

Previous studies have shown that GLA is an effective inhibitor of glioma cell proliferation both *in vitro *and *in vivo *[6,7,14]. During *in vivo *studies infusion of 2 mM GLA at 1 μl/hr for 7 days caused a marked inhibition (~50%) of tumour growth accompanied by apoptosis [7]. This finding stimulated our hypothesis that a longer period of infusion at higher concentrations may be more effective. The present study infused 5 mM GLA at 0.5 μl/hr for 14 days and the tumour area was significantly reduced (75 ± 8.8%) in comparison with the CSF treated tumours and confirmed the efficacy of this treatment method in gliomas.

The presence of GLA caused inhibition of mRNA expression of VEGF, Flt1, ERK1/2 and MMP2 but not Flk1. Immunohistochemical quantification confirmed the decrease in VEGF and Flt1 protein expression and found a lack of change in Flk1 protein content. ERK1 and ERK2 mRNA and protein expression was significantly decreased. The importance of VEGF and its receptors Flt1 and Flk1, along with ERK1/2 and MMP2 in glioma angiogenesis and the inhibitory effects of GLA on these proteins' expression suggested the possibility that angiogenesis could be modified in the GLA-treated tumours. After counting microvessel density (MVD) in both groups it was apparent that GLA had an inhibitory effect on vessel number causing a 44 ± 5.4% reduction in MVD. These interactions are summarized in the scheme presented in Figure 3H. This in itself could be an important mechanism of tumour growth inhibition by reducing tumour nutrition and by reducing the potential for migration along blood vessels whose number is reduced by treatment.

While the role of Flk1 in endothelial proliferation is well known, the role of Flt1 is less well defined. Flt1 can be found at both the cell membrane and in soluble form in the extracellular matrix (ECM) where it is believed to influence the angiogenic process [31,32]. It has been suggested that Flt1 may protect VEGF from proteolytic degradation by binding to it in the ECM [33]. The decrease in Flt1 expression seen in the presence of GLA may reduce the degree of protection of VEGF from degradation in the ECM thereby compounding the effect of decreased VEGF expression. An autocrine loop has been described in neuroblastoma involving VEGF, Flt1 and ERK1/2 [34] and our current data suggest that GLA may interfere with this loop leading to inhibition of tumour cell proliferation and survival in C6 cells *in vivo*.

From the present study it may be suggested that the marked reduction in Flt1 and VEGF expression caused by GLA treatment could cause a reduction in angiogenesis as seen by reduced MVD most likely through reduced endothelial cell proliferation, although this was not quantified directly. The decrease in ERK1 and ERK2 expression may be involved in the decrease in VEGF and Flt1 expression in the GLA treated tumours. In addition, the decrease in ERK1 and ERK2 expression could be directly linked to decreased MMP2 expression and proteolytic activity in the GLA treated tumours. Recent studies have shown that ERK-specific inhibitors cause a decrease in the expression of MMP2 in breast cancer brain metastases [35]. In addition, previous studies have shown that the n-3 PUFA's 20:5 and 22:6 cause decreased pERK concomitant with decreased VEGF expression in HT29 colon cancer cells. These n-3 PUFA's also caused a reduction in HT29 tumour volume *in vivo *and the tumour microvessel density was reduced [36]. It is possible that GLA may affect C6 glioma cells in a similar manner although this remains to be confirmed.

Together with the changes in angiogenesis seen in GLA treated tumours several important changes were found in cell cycle control. The combination of decreased pRb in the absence of functional p16 may cause the cell to lose adequate control over the pRb pathway which is reinforced by the increase in cyclin D1 expression. Increased p53 expression together with these changes in pRb and cyclin D1 can significantly increase cell apoptosis [37-39]. E2F1 expression remained unchanged, in contrast to previous findings *in vitro*, demonstrating the potential importance of the orthotopic tumour microenvironment on cell responses to GLA treatment [9]. The increase in p53 seen in the GLA treated tumour did not lead to altered p21 expression which could explain why pRb was not increased in this model. Instead GLA caused a significant increase in another cyclin-dependent kinase inhibitor, p27, causing a G1/S transition block and subsequently reducing S-phase. This was confirmed by a reduction in S-phase BrdU incorporation and was similar to the findings reported for C6 glioma cells *in vitro *where 150 μM GLA caused a 49% decrease in S-phase [8]. These interactions are summarized in the scheme presented in Figure 3H.

## Conclusion

Overall the findings reported in the present study lend further support to the potential of GLA as an inhibitor of glioma cell proliferation *in vivo *and show it has direct effects upon cell cycle control and angiogenesis in this orthotopic model. Proteins related to angiogenesis which were altered by GLA treatment included VEGF, Flt1, ERK1, ERK2 and MMP2, while those related to cell cycle control altered by GLA treatment included Cyclin D1, pRb, p53 and p27. Combination therapy using drugs with other, complementary targets and GLA could lead to gains in treatment efficacy in this notoriously difficult to treat tumour.

## Competing interests

The authors declare that they have no competing interests.

## Authors' contributions

JAM and MB made an equal contribution to the experimental work reported in this manuscript and as such should be considered as joint first authors. AC participated in the design, execution and analysis of the study and performed the surgical procedures.
